# Forty-ninth Annual Meeting of the Medical Society of the State of New York

**Published:** 1855-03

**Authors:** 


*Forty-ninth Annual Meeting of the Medical Society of the State of New York.*—This society commenced its annual session at the Common Council chamber, on Tuesday, February 6, 1855.

The society was called to order by the President, Dr. Charles B. Coventry, of Utica.

Dr. Coventry then read his inaugural address, mentioning the foundation of the society on the first Tuesday of February, 1807, pursuant to an act entitled, "An Act to Incorporate Medical Societies for the purpose of regulating the Practice of Physic and Surgery in this State, passed April 4th, 1806."

He stated that this was the first legislative acknowledgment of the authority of the profession to examine students and regulate their admission into their own ranks, and the most important act, as regards our profession, ever passed by our Legislature. Before this the law required persons to obtain a license to practice, from a Judge of the Supreme Court.

That the society had done much toward elevating the profession and protecting the community from the evils and impositions of empiricism. That the last year it had succeeded, through the Legislature, in making legal provision for the study of Anatomy.

That, in its published Transactions a valuable collection of facts were to be found. The list of its officers embraced the names of many of the most distinguished in the profession of our state. He said there was an increasing interest manifested in most of the county societies. The action of the Legislature abolishing all penalty for practicing without a license, had by many been supposed equivalent to a repeal of all law regulating our profession. This was a mistaken idea: the action applied not to the profession, but to those out of it; the County Societies retained all the rights and privileges which they ever had, or which pertains to corporate bodies.

The past year witnessed the return of the malignant cholera, followed, as is often the case, by dysentery and fever, but less malignant. This was due in part to the community having become more familiar with the disease, but more to the medical profession, their better knowledge of the means of prevention and treatment.

He alluded to the report of the sanitary commission of New Orleans, as one of the most important events in the medical history of our country for the past year. The commission treated of twenty epidemics of yellow fever, and six of cholera, the first of which extended back to the year 1796, when that city contained but 8756 inhabitants. This report seemed to settle the questions as to the contagious or non-contagious nature of yellow fever and cholera. The report concludes "that yellow fever is not a disease personally contagious, that its infective properties are only communicable in a foul or infected atmosphere." The report gives two practical remarks: first, "that although it is easier to keep from yellow fever than cholera, we can exercise much influence on the causation of both even in their climatic relation; and secondly, "the combination of the terrene and meteorological condition, which is absolutely essential to the existence of either, we certainly have it in our power greatly to control, because by proper policing and regard to other hygienic measures that condition is clearly under our influence."

Communications from various County Medical Societies were received.

Drs. Saunders and Goodrich were appointed committee to invite the medical gentlemen of the Legislature to take seats as honorary members.

Drs. Morrell, Vanderpoel, Carhart, Hoff, Bailey, Quackenbush, Chapin. Douglas and Lansing, were invited to seats as honorary members.

The specimen of extra uterine conception presented by Dr. Parkhurst, was presented by Dr. Armsby carefully prepared. Dr. Armsby read a paper on extra uterine conception, which was referred to committee of publication.

Dr. Phelps, of New York, read a paper on reduction of dislocated femur by manipulation. Dr. Phelps claims this operation as being first performed by Dr. Physick, in 1810—11, at the Pennsylvania Hospital. Referred to committee of publication.

Dr. Monell, of Newburg, presented two specimens of aneurism of the aorta, one of which he deposited with the Albany Medical College for the use of the society. Dr. Monell also read a paper concerning a case of insanity, Referred to committee of publication.

A committee was appointed to solicit the use of the Assembly chamber for the society on Wednesday evening.

Adjourned till 3 o'clock, P. M.

AFTERNOON SESSION.

The society met at 3 o'clock. Minutes of the morning approved.

The Treasurer, Dr. Van O'Linda, submitted his annual report.

From the committee of epidemics a report was made of Orange county, by G. C. Monell. Referred to committee of publication.

Dr. Tilden, by Dr. Armsby, presented a variety of vegetable extracts to the society, for which the thanks of the society were bestowed.

Dr. Griscom presented copies of his anniversary discourse before the N. Y. Academy of Medicine.

Dr. March read misstatements from several medical journals in reference to an operation performed by him about two months since. Also a true state of the case and the causes of death as believed by those physicians who were actors in the scene. 1st. Loss of blood; 2d. Shock to the system; 3d. Introduction of air into the venous circulation.

Dr. Hamilton, of Buffalo, read a paper on the results of dislocations; showing that medical authors, medical men, are not true to others, or even true to themselves, in stating that almost every case of dislocation can be reduced, or that most fractured bones can be made of the same length again. His fracture tables proved part of this statement, and he gave some 130 cases of dislocation, from a number of different surgeons, to establish the other part. Referred to committee of publication.

Dr. March presented the address of Dr. Quackenbush before the Medical Society of the County of Albany.

Adjourned till 10 o'clock, Wednesday, A. M.

SECOND DAY.

Wednesday, A. M.

The society met at 10 o'clock, A. M.

Dr. Snyder read a biographical sketch of the late Dr. Daniel Ayers, of Amsterdam, Montgomery county.

Dr. Coventry read a biographical sketch of the late Prof. James Webster. Dr. Webster was born in Warring, Lancaster, England, in 1793. Graduated at the University of Pennsylvania, 1824. Died at Louisville, Ky., July 18, 1854.

Dr. M'Call, of Utica, read a paper on the needs, duties, and privileges of the medical profession.

Dr. Phelps, of N. Y. city, read a paper on the early and present condition of the medical profession.

It was moved that 3 o'clock be made the hour for the election of officers for the ensuing year.

Dr. Willard, committee on epidemics for the 6th senatorial district, read a paper upon the epidemical diseases of Chenango and Broome counties. Referred to committee of publication.

A communication was received from His Excellency, Gov. Clark, inviting the society to visit him on Thursday afternoon at the Executive Chamber. The invitation was accepted.

Dr. Foster presented a resolution recommending Lunatic Asylum accommodation; 250 as better than those of a larger size, &c. Laid on the table.

Dr. Hamilton resumed his remarks on dislocations. He gave Dr. Nathan Smith, of Mass., the credit of having first reduced the hip by manipulation; he having testified in a malpractice suit giving his mode of reduction. This was before Dr. Physick accidentally reduced the femur at Hospital of Penn.

Dr. Cortiss spoke of fractures of the femur. He said that in adults the femur almost always was fractured obliquely, and in that case there *always* was shortening of the limb.

Dr. March read a paper on operations for harelip.

Adjourned.

AFTERNOON SESSION.

3 o'clock, P. M.

Dr. Staats moved to suspend the order of business, and allow the Rev. Mr. Warren, of the New York Temperance Society, to speak for a few minutes. Mr. Warren asked liberty to interrogate the society on the influences of intoxicating drinks in producing disease, when used continuously in moderate quantities.

Dr. Bradford presented a resolution requesting the society to coöperate in furnishing and diffusing the desired information. Adopted.

The following were elected officers for the ensuing year:

President, Frank H. Hamilton, of Buffalo.
Vice-president, Thomas Hun, of Albany.
Secretary, Howard Townsend, do.
Treasurer, Peter Van O'Linda, do.

The following were elected permanent members:

James R. Wood, B. Fordyce Barker, Philander Stewart, G. C. Monell, Alfred Watkins, Ebenezer Steel, Freeman Turtelot, Henry C. Gray, Luther Guiteau, A. L. Saunders, Marcene Jerry, A. B. Case, John Coventry, Edson Carr, Austin Flint, John B. Coats.

Dr. Horace A. Buttolph, of New Jersey, and Marshall Hall, of London, were elected Honorary members of the society.

Dr. Foster's resolution concerning Lunatic Asylums, was taken up. Drs. Bradford, Cortiss, Staats, Burr, spoke upon the resolution and then it was laid on the table.

Adjourned to the Assembly Chamber.

7 o'clock, P. M.

The society met at the Assembly Chamber, when the President, Dr. Coventry, delivered the Annual Address.

Dr. Collins presented the following:

*Resolved*, That the thanks of this society be tendered to the President for his very interesting and highly valuable address on the present occasion, and that a copy be requested for publication.

The resolution was adopted.

Society adjourned.

This evening Dr. March entertained the society, and several distinguished Albanians, at his own house.

THIRD DAY.

9½ o'clock, A. M.

A letter from Dr. Griscom was read by Dr. Coventry.

J. Kneeland, James L. Phelps, A. Churchill, Eldon Carr, were recommended to the Regents of the University for the Honorary Degree of Doctor of Medicine.

Dr. W. S. Norton read a paper on injury of the shoulder joint.

Dr. Horace Green, of N. Y. city, read a paper on the employment of injections into the bronchial tubes, and into tubercular cavities of the lungs. He claimed to have just performed this operation. Dr. Green read at length the history of some cases that had recovered while under this treatment.

Dr. Foster's resolution, relating to Lunatic Asylums, was taken up and amended as follows:

*Resolved*, That this society is deeply impressed with the importance of further accommodations for the treatment and care of the insane of our state, and they therefore respectfully urge upon the Legislature the speedy passage of the measure now before them for that purpose. Adopted.

Dr. Charles A. Lee was appointed a committee to furnish to the publishing committee an abstract of his review of the trial of John Hendrickson, jr., contained in the American Journal of Med. Science, for 1854.

The thanks of the society were voted to the Common Council and to the Assembly, for the use of their chambers during the session.

Dr. March invited the society to visit the Medical College and the new and beautiful Hospital. For which purpose the society took a recess.

In the afternoon they paid their respects to His Excellency, Governor Clark, at the Executive Chamber.

Adjourned *sine die.*

